# Replication of the Shrimp Virus WSSV Depends on Glutamate-Driven Anaplerosis

**DOI:** 10.1371/journal.pone.0146902

**Published:** 2016-01-11

**Authors:** Chun-Yuan Li, Yi-Jan Wang, Shiao-Wei Huang, Cheng-Shun Cheng, Han-Ching Wang

**Affiliations:** 1 Institute of Biotechnology, College of Bioscience and Biotechnology, National Cheng Kung University, Tainan, Taiwan; 2 Department of Life Science, College of Life Science, National Taiwan University, Taipei, Taiwan; Uppsala University, SWEDEN

## Abstract

Infection with the white spot syndrome virus (WSSV) induces a metabolic shift in shrimp that resembles the “Warburg effect” in mammalian cells. This effect is triggered via activation of the PI3K-Akt-mTOR pathway, and it is usually accompanied by the activation of other metabolic pathways that provide energy and direct the flow of carbon and nitrogen. Here we show that unlike the glutamine metabolism (glutaminolysis) seen in most cancer cells to double deaminate glutamine to produce glutamate and the TCA cycle intermediate α-ketoglutarate (α-KG), at the WSSV genome replication stage (12 hpi), although glutaminase (GLS) expression was upregulated, only glutamate was taken up by the hemocytes of WSSV-infected shrimp. At the same time, we observed an increase in the activity of the two enzymes that convert glutamate to α-KG, glutamate dehydrogenase (GDH) and aspartate aminotransferase (ASAT). α-ketoglutarate concentration was also increased. A series of inhibition experiments suggested that the up-regulation of GDH is regulated by mTORC2, and that the PI3K-mTORC1 pathway is not involved. Suppression of GDH and ASAT by dsRNA silencing showed that both of these enzymes are important for WSSV replication. In GDH-silenced shrimp, direct replenishment of α-KG rescued both ATP production and WSSV replication. From these results, we propose a model of glutamate-driven anaplerosis that fuels the TCA cycle via α-KG and ultimately supports WSSV replication.

## Introduction

Since the early 1990s, white spot disease (WSD) has continued to infect cultured shrimp, and this has led to enormous economic losses [1]. Globally these losses approach $10 billion [2]. Because WSD is considered the most serious shrimp viral disease, and because it has had such a devastating impact on the shrimp aquaculture industry, the World Animal Health Organisation (OIE) has listed WSD as a notifiable crustacean disease. The causative agent of WSD is a novel, large dsDNA virus known as white spot syndrome virus (WSSV) [3]. In order to develop effective anti-WSSV strategies, it is essential to understand the pathogenesis of this unique virus as well as the ways in which it interacts with the host. Recently, by using a systems biology approach, WSSV became the first invertebrate virus known to induce the Warburg effect in infected cells [4, 5].

The Warburg effect, also known as aerobic glycolysis, is the name given to the abnormal glucose consumption and lactate accumulation that occurs under conditions of sufficient oxygen in cancer cells [6]. This effect is also produced by several vertebrate viruses during replication, such as the human cytomegalovirus (HCMV) [7] and human papillomavirus (HPV) [8]. In vertebrates, the metabolic shifts associated with the Warburg effect are accompanied by activation of several biosynthesis pathways, including the pentose phosphate pathway, nucleotide biosynthesis, lipid synthesis and glutaminolysis [9, 10]. Global proteomics and metabolomics studies have shown that the special Warburg effect-like metabolic changes that are seen in WSSV-infected cells during the first WSSV replication cycle (22–24 hpi), are likewise associated with changes that lead to the production of both energy and the precursors of macromolecular biosynthesis [5]. It has also been established that the WSSV-induced Warburg effect is required for completing WSSV replication [4, 5, 11].

**Fig 1:**
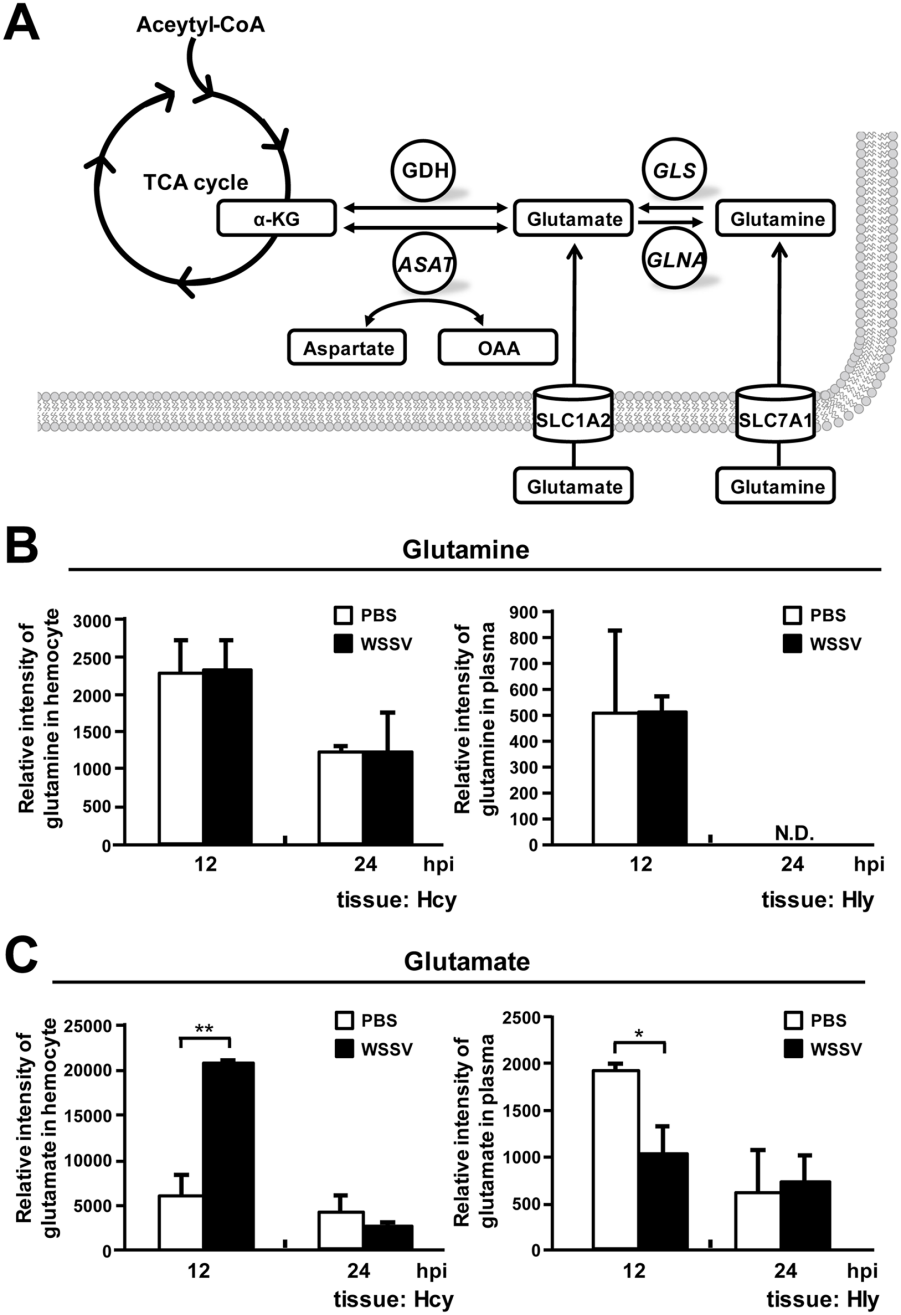
The glutaminolysis pathway is driven by the uptake of glutamate at 12 hpi. (A) Simplified schematic of the glutaminolysis metabolic pathway. Abbreviations: GLS, glutaminase; GLNA, glutamine synthetase; GDH, glutamate dehydrogenase; ASAT, aspartate aminotransferase; α-KG, α-ketoglutarate; OAA, oxaloacetate; SLC1A2: transporter involved in glutamate transport; SLC7A1: transporter involved in glutamine transport. Relative intensity of (B) glutamine and (C) glutamate was measured by LC-ESI-MS at the WSSV replication stage (12 hpi) and late stage (24 hpi). Samples of hemocytes (Hcy) and hemolymph (Hly) were collected at the indicated time points. Quantitative metabolomic LC-ESI-MS data were collected from a single replication of 5–6 samples of 10 shrimp each. (A summary of the hemocyte data appeared previously in Su *et al*. [5]. Hemolymph data is from the same metabolomic study, but has not been previously published.) Asterisks indicate statistically significant differences between the PBS control and WSSV infection groups (Student’s t test, * *p*<0.05, ** *p*<0.01).

Two of the most important carbon sources used by mammalian cells are glucose and glutamine. Under the Warburg effect, glucose is converted to pyruvate and is then diverted into lactate production, rather than entering the tricarboxylic acid (TCA) cycle [12, 13, 14]. In cancer cells, however, the TCA cycle is maintained by exogenous glutamine, which is used as an alternative carbon source that replenishes TCA cycle intermediates (anaplerosis) via glutamine metabolism (glutaminolysis or glutamine-driven anaplerosis) [13, 15, 16]. The key intermediate in this process is α-ketoglutarate (α-KG): glutaminase (GLS) converts glutamine into glutamate, which in turn is converted into α-KG by glutamate dehydrogenase (GDH) and/or aspartate aminotransferase (ASAT) (Fig 1A; [17]). Anaplerotic fill of the TCA cycle by α-KG allows the cycle to keep functioning even under conditions of aerobic glycolysis, and this provides NADH for oxidative phosphorylation as well as intermediates that serve as precursors for biosynthesis [15], such as aspartate for nucleotide synthesis and citrate for *de novo* lipid synthesis [18, 19, 20]. The importance of the TCA cycle and the need for it to keep functioning explains why most cancer cells undergoing the Warburg effect show a dramatic increase in glutaminolysis [15, 18]. In the present paper, we investigate whether similar changes in glutaminolysis also occur during the Warburg-like effect that is induced by WSSV in infected shrimp cells and if so how these metabolic alterations might benefit WSSV genome replication.

There is already preliminary evidence for these changes: in a previous study of the metabolome of shrimp hemocytes, we found that during the WSSV-induced Warburg effect at the WSSV genome replication stage (12 hpi), there was also an accumulation of both glutamate and of the TCA cycle intermediates that follow α-KG [5]. In the first part of this study, by using a previously constructed metabolic database compiled from ESI-LC-MS data obtained as described in Su *et al*. [5], we identified and measured the levels of metabolites involved in glutamine metabolism in shrimp hemocytes and hemolymph at 12 and 24 hpi. After finding that WSSV triggers glutamate-driven anaplerosis at 12 hpi, in the second part of this study, we investigated the expression of glutaminolysis-related genes, and then, since the PI3K-Akt-mTOR pathway is known to be involved in the regulation of the Warburg effect [5, 12, 21], we used a series of inhibitor experiments to determine whether this pathway and the mTORC2 pathway are also involved in regulating WSSV-induced glutamate-driven anaplerosis. Lastly, we used dsRNA silencing and exogenous α-ketoglutarate replenishment to confirm that glutamate-driven anaplerosis is essential for WSSV replication. From our findings, we propose a model of how WSSV-triggered glutamate-driven anaplerosis–rather than the much more usual glutamine-driven glutaminolysis–acts to replenish the TCA cycle during the WSSV genome replication stage.

## Materials and Methods

### Experimental animals and virus inoculum

Batches of *Litopenaeus vannamei* shrimp (~3g of body weight) were purchased from the Aquatic Animal Center, National Taiwan Ocean University (NTOU), and maintained for 1~3 days in water tank systems containing sterilized seawater (30 ppt at 25~27°C). White spot syndrome virus (WSSV) (Taiwan isolate, GenBank accession no. AF440570) was used for all the challenge experiments. For the preparation of WSSV stock, hemolymph was collected from WSSV-infected moribund shrimp and centrifuged at 4°C at 10,000 g for 10 min. The supernatant containing the WSSV was collected and diluted by using 1x phosphate-buffered saline (PBS) (137 mM NaCl, 2.7 mM KCl, 10 mM Na_2_HPO_4,_ 2 mM KH_2_PO_4_) at a ratio 1:4 and then stored at −80°C before use. The final inoculums were prepared from the virus stock by 10,000x dilution with 1x PBS before being used. An IQ REAL kit was sued to analyze aliquots of the diluted inoculum, and the viral copy number was found to be 2.2 copies/μl. Each shrimp received a 100 μl WSSV inoculum (i.e. 220 WSSV copies), a dosage which caused approximately 50% mortality at 72h post injection.

### Using a previously compiled database of liquid chromatography electrospray ionization mass spectrometry (LC-ESI-MS) data to identify the changes of glutamine and glutamate in hemocytes and hemolymph collected from WSSV-infected shrimp

In a previous study, we used LC-ESI-MS to measure the metabolic changes induced by WSSV in hemocytes and hemolymph collected from WSSV-infected shrimp that had been pretreated with the mTOR inhibitor Torin1 or with PEG solvent (0.25% polyethylene glycol, 0.25% Tween 20 and 0.15 M NaCl) [5]. These results were subsequently compiled into a metabolomic database that we used here to analyze changes in the levels of glutamine and glutamate during WSSV replication. Two hours after pretreatment with Torin1 or PEG solvent, the shrimp in this experiment were challenged with WSSV or PBS by intramuscular injection, and at 12 and 24 hpi, hemocytes and hemolymph were collected using an anticoagulant (450 mM NaCl, 10 mM KCl, 10 mM EDTA, 10 mM Tris-HCl, pH 7.5). From each experimental group, 4–5 shrimp samples were then prepared (10 shrimp in each sample) and used for LC-ESI-MS metabolic analysis as described in our previous study [5].

### Effect of the inhibitors Torin1, LY294002 and Rapamycin on the mRNA expression of glutaminolysis-related genes

Since all of the hemocyte and hemolymph samples taken from the above Torin1 and PEG only (control) groups were used for metabolomics analysis, pleopod samples (4–5 samples from each group; 10 pleopods in each sample) were also collected from these groups.

To evaluate the involvement of the PI3K-mTORC1 pathway, 10 individual hemocyte samples were collected from other groups of WSSV-infected shrimp that had been pretreated by injection with 100 μl of the inhibitor LY294002 (dissolved in 10% DMSO and diluted with PBS; 0.625 μg/g shrimp) or with vehicle only (0.01% DMSO; diluted with PBS). WSSV challenge by injection with 100 μl of virus inoculum was performed 2 hours after this pretreatment, and the hemocyte samples were collected at 12 and 24 hpi.

Lastly, to investigate the effect of inhibiting mTORC1, groups of shrimp were pretreated with Rapamycin (0.02 μg/g shrimp) or PEG solvent by injection 2 h before being challenged with WSSV. At 12 and 24 h after WSSV challenge, four hemocyte samples were collected from the shrimp in each group, with each sample being taken from 3 shrimp.

For all of the above samples, the mRNA expression levels of the enzymes GDH, ASAT and GLS were determined by real-time PCR as described below.

### Measurement of GDH activity after WSSV infection

Hemocytes from shrimp injected with WSSV or PBS (5 samples from each group, with hemocytes from 3 shrimp in each sample) were collected at 12 and 24 hpi. The hemocyte samples were homogenized by using plastic sticks in microtubes in 200 μl of the ice-cold GDH assay buffer supplied with a commercial glutamate dehydrogenase activity colorimetric assay kit (Biovision) and then centrifuged at 4°C (13,000 g for 10 min) to remove the cell debris. Protein concentrations in each sample were measured using a Bio-Rad protein assay, and approximately 20 μg of protein from each sample was placed in the wells of a 96-well plate with the final volume adjusted to 50 μl/well by adding GDH assay buffer. These 50 μl lysate samples as well as the standards supplied with the GDH activity kit (50 μl/well) were then mixed with a 100 μl reaction mixture containing 82 μl GDH assay buffer, 8 μl GDH developer and 10 μl 2M glutamate. The samples were incubated at 37°C for 95 min (T1), and the absorbance of each sample was then measured at 450 nm to give the A_n_ value. After incubating for another 30 min (for a total of 125 min [T2] of incubation), the absorbance of each sample was measured again at 450 nm to give the A_n+1_ value. After the difference in absorbance (A_n+1_ –A_n_) was converted to the NADH amount (B) by using an NADH standard curve, the GDH activity was then calculated as follows: GDH activity (mU/mg) = B / ([T2-T1] × 20 μg). Statistically significant differences between groups were analyzed by Student's *t*-test.

### Measurement of ASAT activity after WSSV infection

Hemocytes from shrimp injected with WSSV or PBS (4 samples from each group, with hemocytes from 4 shrimp in each sample) were collected at 12 and 24 hpi. The hemocyte samples were homogenized by using plastic sticks in a microtubes in 200 μl of the ice-cold AST assay buffer supplied with a commercial aspartate aminotransferase (AST, ASAT or SGOT) activity colorimetric assay kit (Biovision) and then centrifuged at 4°C (13,000 g for 10 min) to remove the cell debris. Protein concentrations in each sample were measured using a Bio-Rad protein assay, and approximately 1 μg of protein from each sample was placed in the wells of a 96-well plate with the final volume adjusted to 50 μl/well by adding AST assay buffer. These 50 μl lysate samples as well as the standards and positive control supplied with the AST activity kit (50 μl/well) were then mixed with a 100 μl reaction mixture containing 80 μl AST assay buffer, 2 μl AST enzyme mix, 8 μl developer and 10 μl AST substrate. The samples were incubated at 37°C for 140 min (T1), and the absorbance of each sample was then measured at 450 nm to give the A_n_ value. After incubating for another 60 min (for a total of 12 hr [T2] of incubation), the absorbance of each sample was measured again at 450 nm to give the A_n+1_ value. After the difference in absorbance (A_n+1_ –A_n_) was converted to the glutamate amount (B) by using an glutamate standard curve, the ASAT activity was then calculated as follows: ASAT activity (mU/mg) = B / ([T2-T1] × 1 μg). Statistically significant differences between groups were analyzed by Student's *t*-test.

### Determination of the concentration of α-ketoglutarate levels after WSSV infection

At 12 and 24 h after injection with WSSV or PBS, shrimp stomachs were collected (5 samples from each group at each time point, with 10 shrimp stomachs in each sample), and an Alpha-Ketoglutarate Colorimetric/Fluorometric Assay Kit (Biovision) was used to measure the ketoglutarate levels according to the manufacturer’s instructions. Samples of approximately 30 mg were placed in 150 μl of α-KG assay buffer and homogenized by using plastic sticks in microtubes. Proteins and insoluble debris were then removed by using 10 kDa molecular weights cut off spin columns. Using a 96-well plate system, lysate samples (40 μl per well) were added into duplicate wells (one for α-KG measurement and another one for background control) and brought to a final volume of 50 μl with α-KG assay buffer. For the α-KG measurement, each well was mixed with a reaction mixture (50 μl) consisting of 44 μl α-KG assay buffer, 2 μl α-KG converting enzyme, 2 μl enzyme mix and 2 μl probe. For the background control, in order to subtract the signal due to the pyruvate that was already present, 50 μl of the reaction mixture without the α-KG converting enzyme (and with two additional μl of α-KG assay buffer) was used instead. Incubation proceeded at 37°C for 30 min in the dark, and the absorbance of each sample was measured at 570 nm. After subtraction of the background value, the corrected OD_570_ values were applied to the standard curve to get the amount of α-KG (*x* nmol). The α-KG concentrations were then calculated as follows: α-KG concentration (mM) = *x*/40 μl. Statistically significant differences between groups were analyzed by Student's *t*-test.

### *In vivo* silencing of LvGDH and LvASAT expression by dsRNA mediated RNA interference

The dsRNAs were prepared as described in our previous study [11]. As is common practice in shrimp silencing studies, EGFP dsRNA was used as the non-specific control. Briefly, partial sequences of GDH, ASAT and the EGFP control were amplified by PCR with the following primer sets, respectively: GDH-F/GDH-R, ASAT-F/ASAT-R and EGFP-F/EGFP-R (Table 1). T7 promoter sequence was then added to the 5’ and 3’ ends of these PCR amplicons by performing PCR with the following primer sets: GDH: T7-GDH-F/GDH-R and GDH-F/T7-GDH-R; ASAT: T7-ASAT-F/ASAT-R and ASAT-F/T7-ASAT-R; EGFP control: T7-EGFP-F/EGFP-R and EGFP-F/ T7-EGFP-R (Table 1). After the ssRNAs were synthesized by using the T7 RiboMAX Express large-scale RNA production system (Promega), the corresponding pairs of ssRNAs were mixed gently, incubated at 70°C for 20 min and cooled down to room temperature for 30 min to form the dsRNAs. Next, each dsRNA was purified by the phenol/chloroform/isoamyl alcohol method. The quantity and quality of each dsRNA was measure by UV spectrophotometer and agarose gel electrophoresis, respectively. The specific silencing ability of the GDH dsRNA was confirmed by Western blotting (S1 Fig). (Unfortunately this method could not be used for ASAT dsRNA because a suitable antibody is not available.) The dsRNA products were stored at -80°C until used for the following *in vivo* silencing experiment.

**Table 1:** PCR primers used in this study.

Gene	Primer name	Primer sequence (5’-3’)	Usage
*Lv*GDH			
	GDH-F	5’-GATTGGTATCACTCCGGGAT-3’	Cloning of the partial cDNA
	GDH-R	5’-GACACAGTTACACCACCAG-3’	Cloning of the partial cDNA
	T7-GDH-F	5’-TAATACGACTCACTATAGGGAGAGATTGGTATCACTCCGGGAT-3’	dsRNA synthesis
	T7-GDH-R	5’-TAATACGACTCACTATAGGGAGAGACACAGTTACACCACCAG-3’	dsRNA synthesis
	GDH-real-F	5’-TGAGGAGAAGCGCAACAAGA-3’	Real-time PCR
	GDH-real-R	5’-TGGCAGGGCTCCATGATC-3’	Real-time PCR
*Lv*ASAT			
	LvASAT-2-real F	5’- CGCTTCGCTCTGCCAACT -3’	Real-time PCR
	LvASAT-2-real R	5’- CGACGGCCATTTCGAAAA -3’	Real-time PCR
	ASAT-F	5’- CGCGGAAGGAATACGCTTC-3’	Cloning of the partial cDNA
	ASAT-R	5’- CGCCAAGTNCCAGAAAAACC -3’	Cloning of the partial cDNA
	T7-ASAT-F	5’- TAATACGACTCACTATAGGGAGACGCGGAAGGAATACGCTTCG -3’	dsRNA synthesis
	T7-ASAT-R	5’- TAATACGACTCACTATAGGGAGACGCCAAGTNCCAGAAAAACC -3’	dsRNA synthesis
*Pm*SLC7A1			
	SLC7A1-real-F	5’- GGTGCCCGTGTACCAAAGG -3’	Real-time PCR
	SLC7A1-real-R	5’- TCGCCAACGCATACATAGCT -3’	Real-time PCR
*Pm*SLC1A2			
	SLC1A2-real-F	5’- GGGCTCCTCAGGCAAGATG -3’	Real-time PCR
	SLC1A2-real-R	5’- AGCCAGAATAGTGGTGGAGAAGTAA -3’	Real-time PCR
*Pm*GLS			
	GLS-real-F	5’- CGTCAGGGACGTCCTCT -3’	Real-time PCR
	GLS-real-R	5’- CGAGTAGTCGTACATGC -3’	Real-time PCR
EGFP			
	EGFP-F	5’-GTTCAGCGTGTCCGGCGAG-3’	dsRNA synthesis
	EGFP-R	5’-GTTCTTCTGCTTGTCGGCC-3’	dsRNA synthesis
	T7-EGFP-F	5’-TAATACGACTCACTATAGGGAGAGTTCAGCGTGTCCGGCGAG -3’	dsRNA synthesis
	T7-EGFP-R	5’-TAATACGACTCACTATAGGGAGAGTTCTTCTGCTTGTCGGCC -3’	dsRNA synthesis
EF1α			
	EF1α-real-F	5’-ACGTGTCCGTGAAGGATCTGAA-3’	Real-time PCR
	EF1α-real-R	5’-TCCTTGGCAGGGTCGTTCTT-3’	Real-time PCR
WSSV VP28			
	VP28-real-F	5’-AGTTGGCACCTTTGTGTGTGGTA-3’	Real-time PCR
	VP28-real-R	5’-TTTCCACCGGCGGTAGCT-3’	Real-time PCR
Others			
	Anchor-dTv	5’-GACCACGCGTATCGATGTCGACTTTTTTTTTTTTTTTTV-3’	cDNA synthesis
	M13F	5’- GTTTTCCCAGTCACGAC -3’	colony PCR check
	M13R	5’-TCACACAGGAAACAGCTATGAC-3’	colony PCR check

The added T7 promoter sequence is underlined.

Shrimps (3g mean body weight) in this experiment were injected with LvGDH dsRNA, LvASAT dsRNA, or EGFP dsRNA (control) at a concentration of 0.1 μg/g shrimp. Three days after the dsRNA injection, the shrimp were subjected to challenge with WSSV by injection with 100 μl of virus inoculum. At 24 hpi, 4 samples of hemocytes and pleopods (3 shrimp in each sample) were collected from each group. The hemocyte samples were used to measure the host gene expression while the pleopod samples were used to measure the WSSV genome copy number.

### Replenishment of α-ketoglutarate

Three days after LvGDH dsRNA injection and 2 h after WSSV challenge, shrimp (~3g) were injected with dimethyl-α-oxoglutarate (α-KG; Sigma-Aldrich; 531 μg α-KG/g shrimp). At 24 hpi, four samples of hemocytes and pleopods (3 shrimp per sample) were collected. The ATP/ADP ratio in the hemocyte samples was measured as described below, and the gene expression of GDH and WSSV VP28 was measured by using real-time PCR. The IQ Real^™^ WSSV quantitative system (GeneReach Biotechnology Corp.) was used to measure the WSSV genome copy number in the pleopod samples. Statistically significant differences between the LvGDH experimental group and the EGFP non-specific control group were analyzed by Student's *t*-test.

### Quantification of host genes and the WSSV viral gene VP28 by real-time PCR

Total RNA was extracted from the shrimp samples and subjected to cDNA synthesis using Superscriptase II Reverse Transcriptase (Invitrogen) with Anchor-dTv primer (Table 1). The cDNA samples were then stored at -20°C until used to determine the mRNA expression of the respective target genes by real-time PCR.

The stored samples of cDNA were subjected to real-time PCR using the Bio-Rad detection system with KAPA SYBR^®^ FAST Master Mix (KAPA) to measure the relative expression levels of the host genes GDH, ASAT, GLS, SCL7A1, SLC1A2 and EF1α as well as the highly expressed WSSV structural gene VP28. Specific primer sets used in this study are listed in Table 1. As EF1α was used as an internal control [22], data values were normalized to EF1α cDNA and calculated by the 2^-ΔΔCT^ method. Statistically significant differences between groups were analyzed either by Student's *t*-test, or else, if the distribution of the samples was non-normal, by the Mann-Whitney U test.

### Quantification of the WSSV genome copy number

The pleopod samples from the silencing experiment were subjected to genomic DNA extraction using a DTAB/CTAB DNA extraction kit (GeneReach Biotechnology Corp.), and the copy numbers of WSSV genomic DNA were quantified by the IQ Real^™^ WSSV quantitative system (GeneReach Biotechnology Corp.). Statistically significant differences between groups were analyzed by Student's *t*-test.

### Determination of ATP/ADP ratio

The ATP/ADP ratio in the hemocyte samples collected from the α-KG replenishment experiment was determined with an ApoSENSOR^™^ ADP/ATP Ratio Bioluminescent Assay Kit (Biovision) using a slightly modified protocol as described in a previous study [4]. For each sample in a 96-well plate, 10 μl of hemocytes were mixed with 100 μl Nucleotide Releasing Buffer and shaken for 5 min. After adding 1 μl ATP Monitoring Enzyme to each well and allowing the reaction to proceed for 2 min, the luminescence (B) was determined by using a luminometer. After 10 min, the luminescence was recorded again to provide another data point (C). Next, to measure the ADP level in the cell, 1 μl ADP Converting Enzyme was added into each well and the luminescence (D) was measured again 2 min later. For the background control, 10 μl PBS was subjected to the same procedure to get the corresponding data point B’, C’ and D’. The ATP/ADP ratio was then calculated as: (B–B’)/([D–D’]–[C–C’]).

## Results

### Metabolomic analysis of glutamine and glutamate in shrimp hemocytes and hemolymph during the WSSV replication cycle

During glutaminolysis, glutamine is first metabolized into glutamate and the resulting glutamate is then converted into α-ketoglutarate, which is an intermediate that replenishes the TCA cycle (Fig 1A). A previous LC-ESI-MS quantitative metabolomics study showed that WSSV infection led to changes in the levels of glutaminolysis-related amino acids in shrimp hemocytes [5]. Although the levels of glutamine in the hemocytes and plasma were not affected at either the WSSV genome replication stage (12 hpi) or the late stage (24 hpi) of the WSSV replication cycle (Fig 1B), at the WSSV genome replication stage (12 hpi), the level of glutamate was increased in hemocytes and decreased in plasma (Fig 1C). These results suggest that WSSV infection may induce the uptake of glutamate from the hemolymph into the hemocytes, which may in turn help to drive glutamate-driven anaplerosis, as opposed to glutamine-driven anaplerosis (glutaminolysis).

### Additional evidence that WSSV infection activates glutamate-driven anaplerosis at the genome replication stage of the WSSV replication cycle (12 hpi)

**Fig 2:**
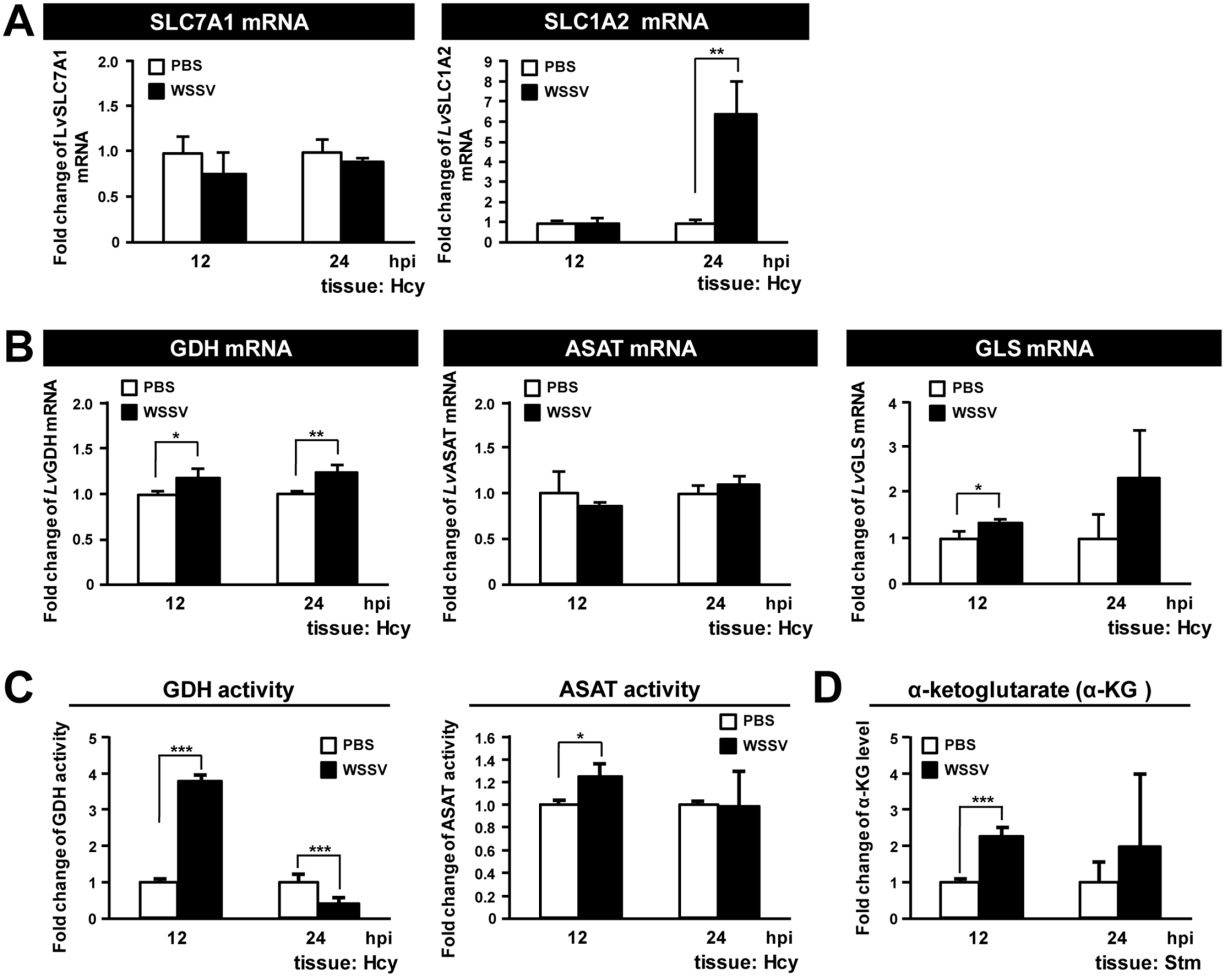
WSSV induces glutamate dehydrogenase (GDH) expression at the WSSV replication stage (12 hpi). (A) The mRNA level of SLC7A1 and SLC1A2 during WSSV infection. Gene expression in hemocyte samples (4 samples for each group at each time point; 4 shrimp in each sample) was analyzed by real-time PCR (qPCR). (B) The mRNA level of GDH, ASAT and GLS during WSSV infection. Gene expression in hemocyte samples (4 samples for each group at each time point; 4 shrimp in each sample) was analyzed by real-time PCR (qPCR). (C) Analysis of lysates from hemocytes collected at 12 and 24 hpi showed that the activities of GDH and ASAT, the key enzymes involved in the conversion of glutamate to α-ketoglutarate (α-KG), were upregulated at the WSSV genome replication stage (12 hpi). The values are shown as fold change compared to the PBS group. (D) Analysis of lysates from stomachs collected at 12 and 24 hpi showed that the level of α-KG was increased at 12 hpi. (Stomachs were used for this analysis because lysates from hemocytes did not contain detectable levels of α-KG.) Each bar represents the mean ± SD. Asterisks indicate statistically significant differences between WSSV infection groups and the corresponding PBS control (Student’s t test, * *p*<0.05, ** *p*<0.01, *** *p*<0.001).

To further elucidate how WSSV infection impacts glutaminolysis, we next investigated WSSV-induced changes in the gene expression of 5 glutaminolysis-related genes in the hemocyte of infected shrimp. For the glutamine transporter SLC7A1 [23], mRNA levels were unaffected by WSSV, while for the glutamate transporter SLC1A2 [24], WSSV induced significant mRNA upregulation only at 24 hpi (Fig 2A). The mRNA levels of the enzyme GDH were significantly increased at both 12 and 24 hpi (Fig 2B), and GDH activity was also increased at 12 hpi (Fig 2C). Although GDH activity was subsequently reduced, the 12 hpi data is consistent with our previous finding that WSSV infection induced increased protein levels of GDH in hemocytes [5]. Fig 2B also shows that ASAT mRNA levels were unchanged and that GLS mRNA levels were significantly increased only at 12 hpi. However, the enzyme activity of ASAT was significantly enhanced in shrimp hemocytes at the WSSV genome replication stage (Fig 2C), and we further note that ASAT protein levels were also elevated in WSSV-infected shrimp (unpublished data). In stomach cells, production of a key intermediate in glutaminolysis, α-KG, was also significantly increased at the WSSV genome replication stage (Fig 2D). (Stomach tissue, which is another main target organ of WSSV and also shows the WSSV-induced Warburg effect, was used instead of hemocytes in this assay because it is difficult to obtain a sufficient quantity of hemocytes for an α-KG determination.)

### mTORC2 is a positive regulator of GDH gene expression at 12 hpi

**Fig 3:**
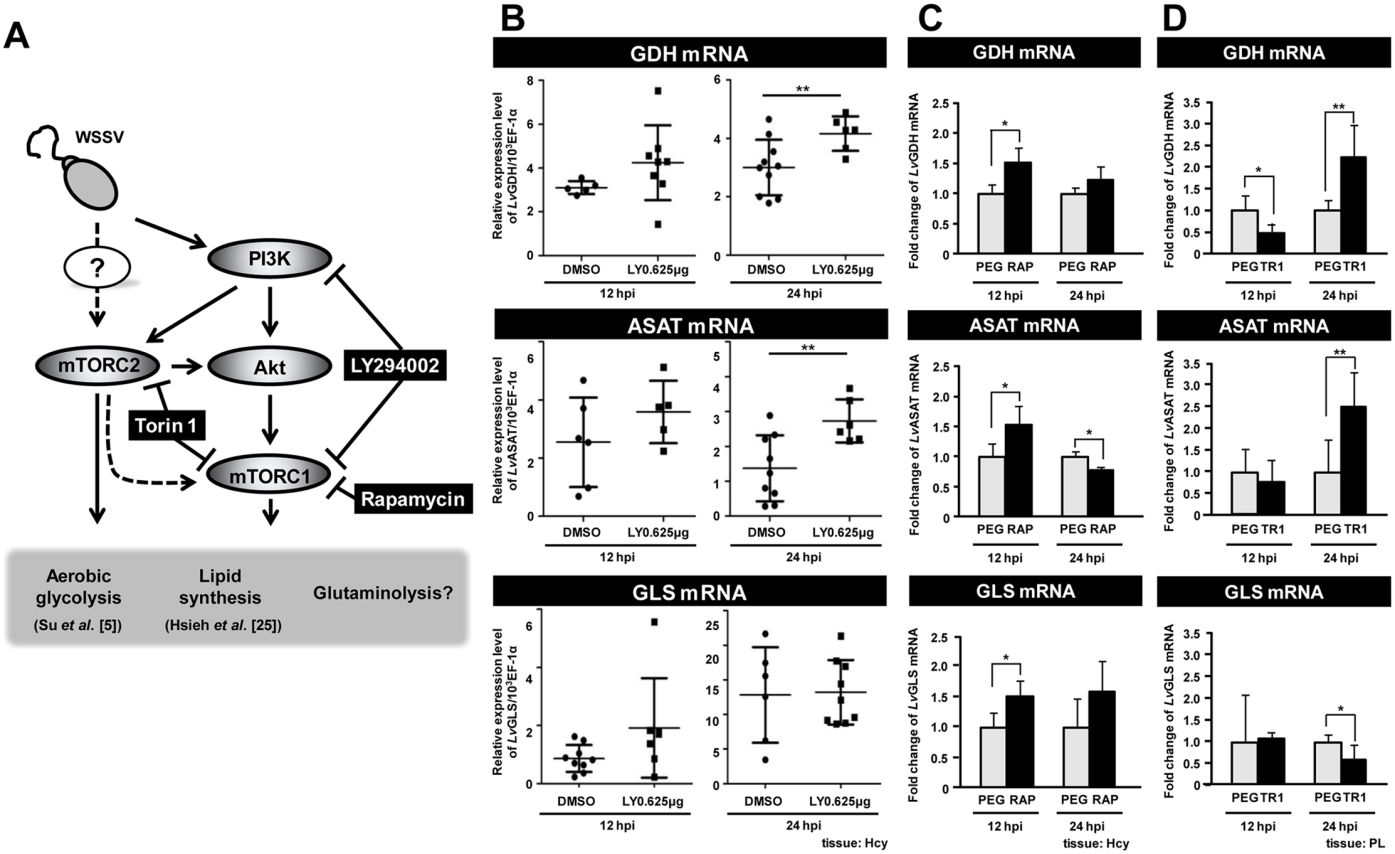
WSSV-induced expression of GDH is regulated by mTORC2. (A) Schematic of the PI3K-Akt-mTOR pathway and the three inhibitors used in this experiment (black boxes). All of the results shown in this Figure are from WSSV-infected shrimp, i.e. the controls were not treated with the various inhibitors, but they were infected. (B) Real-time PCR (qPCR) analysis shows that the PI3K/mTORC1 inhibitor LY294002 had no significant effect on WSSV-induced GDH expression at 12 hpi. Gene expression was measured in 10 individual hemocyte samples for each group at each time point. (The number of data points shown may be less than 10 because any sample that failed to produce a sufficiently specific Bio-Rad amplification plot was rejected.) Bars represent the mean ± SD. Asterisks indicate statistically significant differences in WSSV-infected shrimp between the drug treatment groups and the corresponding vehicle-only control. The Mann-Whitney U test was used to test the non-normal LY294002 12 hpi GLS group data against the DMSO control. Student’s *t*-test was used for all other comparisons (** *p*<0.01). (C) Inhibition of mTORC1 by Rapamycin significantly up-regulated the expression of GDH, ASAT and GLS mRNA at 12 hpi. Gene expression in hemocyte samples (4 samples for each group at each time point; 3 shrimp in each sample) was analyzed by real-time PCR (qPCR). Asterisks indicate statistically significant differences in WSSV-infected shrimp between the drug treatment groups and the corresponding vehicle-only control (Student’s t test, * *p*<0.05, ** *p*<0.01). (D) Inhibition of mTORC1/C2 by Torin 1 significantly suppressed the expression of GDH, but no effect on ASAT and GLS at 12 hpi. Gene expression in pleopod samples (5–6 samples for each group at each time point; 10 shrimp in each sample) was analyzed by real-time PCR (qPCR). Each bar represents the mean ± SD. Asterisks indicate statistically significant differences in WSSV-infected shrimp between the drug treatment groups and the corresponding vehicle-only control (Student’s t test, * *p*<0.05, ** *p*<0.01).

In vertebrates, activation of the PI3K-Akt-mTOR pathway has been shown to trigger the Warburg effect and the metabolic events associated with the Warburg effect [9, 17]. During WSSV infection, activation of this pathway has likewise been shown to be important for triggering the Warburg effect at 12 hpi [5] and for lipogenesis at 24 hpi [25] (Fig 3A). However, it remains unknown if the PI3K-Akt-mTOR pathway also acts to regulate glutamate-driven anaplerosis.

To examine this possibility, we first treated shrimp with LY294002, which inhibits PI3K and one of the mTOR complexes (mTORC1). As shown in Fig 3B, at the WSSV genome replication stage, compared to the group treated with adjuvant only (0.01% DMSO in PBS), LY294002 had no significant effect on the mRNA expression of GDH, ASAT or GLS. However, at 24 hpi, suppression of the PI3K- mTORC1 pathway led to up-regulation of GDH and ASAT mRNA, which suggests that this pathway might suppress the expression of these molecules at the late stage of WSSV infection.

In our next experiment, we treated shrimp with Rapamycin, which inhibits mTORC1. As shown in Fig 3C, at the WSSV genome replication stage, Rapamycin treatment enhanced the expression levels of all three enzymes, which suggests that mTORC1 acts as an inhibitor of GLS, GDH and ASAT. However, we note that since mTORC2 can sometimes become activated when mTORC1 is suppressed [26], then it may be that mTORC2 is driving the expression of these enzymes.

To investigate this possibility, we therefore used Torin 1 to suppress both of these mTOR complexes. At 12 hpi, Torin 1 treatment led to a significant decrease of GDH, but had no effect on the levels of ASAT or GLS (Fig 3D). Taken together, these data suggest that, at 12 hpi, the WSSV-induced expression of GDH is predominantly regulated by mTORC2, and not by the PI3K-mTORC1 pathway. Conversely, at 24 hpi, PI3K and mTORC2 seem to act as negative regulators that suppresses the expression of at least two glutaminolysis-related genes (GDH and ASAT). However, this suppression is evidently not mediated via the PI3K-mTORC1 pathway. Lastly we note that the treatments with LY294002 (Fig 3B) and Rapamycin (Fig 3C) both lead to an increase in GLS expression at 12 hpi. Although the increase was statistically significant only in the Rapamycin group, taken together, these results suggest that the PI3K-mTORC1 pathway may be acting to suppress GLS expression at this time. (We note that, pleopod samples were used in Fig 3D is because pleopods and hemocytes are all WSSV-target tissues and both cell types show a similar WSSV replication cycle [4]. However, we can not exclude the any possibility that some of gene responses in pleopods we observed may be differ with hemocytes. The further investigation may be needed).

### GDH and ASAT are both important for WSSV replication

**Fig 4:**
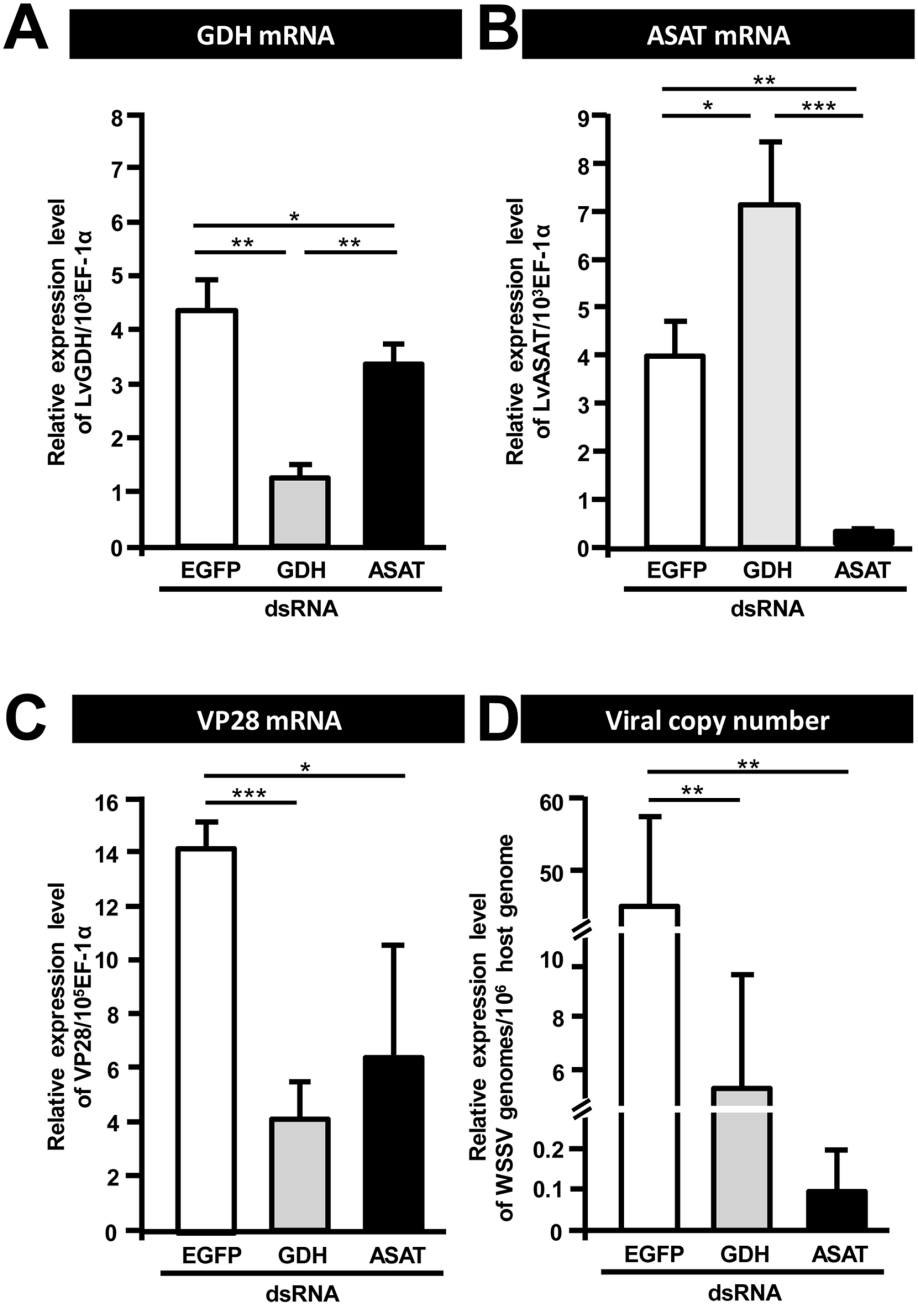
GDH and ASAT are involved in WSSV replication. Three days after injection of the indicated dsRNA, shrimp were challenged with WSSV by injection, and 24h later, 4 hemocyte and pleopod samples (3 shrimp in each sample) were collected from each group. The hemocyte samples were used to measure mRNA expression while the pleopod samples were used to measure the viral copy number. *In vivo* gene silencing of (A) GDH and (B) ASAT by injection of corresponding dsRNA induced a significantly stronger decrease in the respective target mRNA expressions compared to the groups treated with non-specific EGFP dsRNA. *In vivo* gene silencing of GDH and ASAT decreased both (C) WSSV gene VP28 expression and (D) WSSV genome copy numbers at 24 h post WSSV injection. Asterisks indicate statistically significant differences between EGFP dsRNA control, GDH dsRNA and ASAT dsRNA groups (Student’s t test, * *p*<0.05, ** *p*<0.01, *** *p*<0.001).

To investigate the importance of GDH and ASAT for WSSV replication, shrimp were treated with the corresponding dsRNAs to silence the expression of these two enzymes. Shrimp were challenged with WSSV 3 days post dsRNA injection and hemocytes and pleopods were collected at 24 h post WSSV injection. Relative to the non-specific dsRNA injection group, both GDH and ASAT mRNA were significantly decreased by their corresponding dsRNAs (Fig 4A & 4B). Fig 4C and 4D further show that in the groups of shrimp pretreated with GDH dsRNA and ASAT dsRNA, the WSSV VP28 expression levels and WSSV viral copy numbers were significantly inhibited compared to the EGFP dsRNA-treated group. Together, these data suggest that both GDH and ASAT are important for WSSV replication.

### The effect of GDH silencing in WSSV-infected shrimp can be rescued by injection of the TCA cycle intermediate α-KG

The above results suggest that in WSSV-infected shrimp, glutamate replenishes the TCA cycle via conversion to α-KG through GDH and ASAT. If so, then after GDH silencing, it should be possible to rescue virus replication by directly supplying the infected shrimp with an additional amount of the TCA cycle intermediate α-KG. To assess this possibility, GDH silenced shrimp were treated with α-KG (531 μg/g) or PBS only 2 h after WSSV injection. Since any difference in WSSV mRNA and virus copy number would only be apparent at the late stage of the infection cycle (i.e. 24 hpi), samples of hemocytes and pleopods were collected at 24 h post WSSV injection.

**Fig 5:**
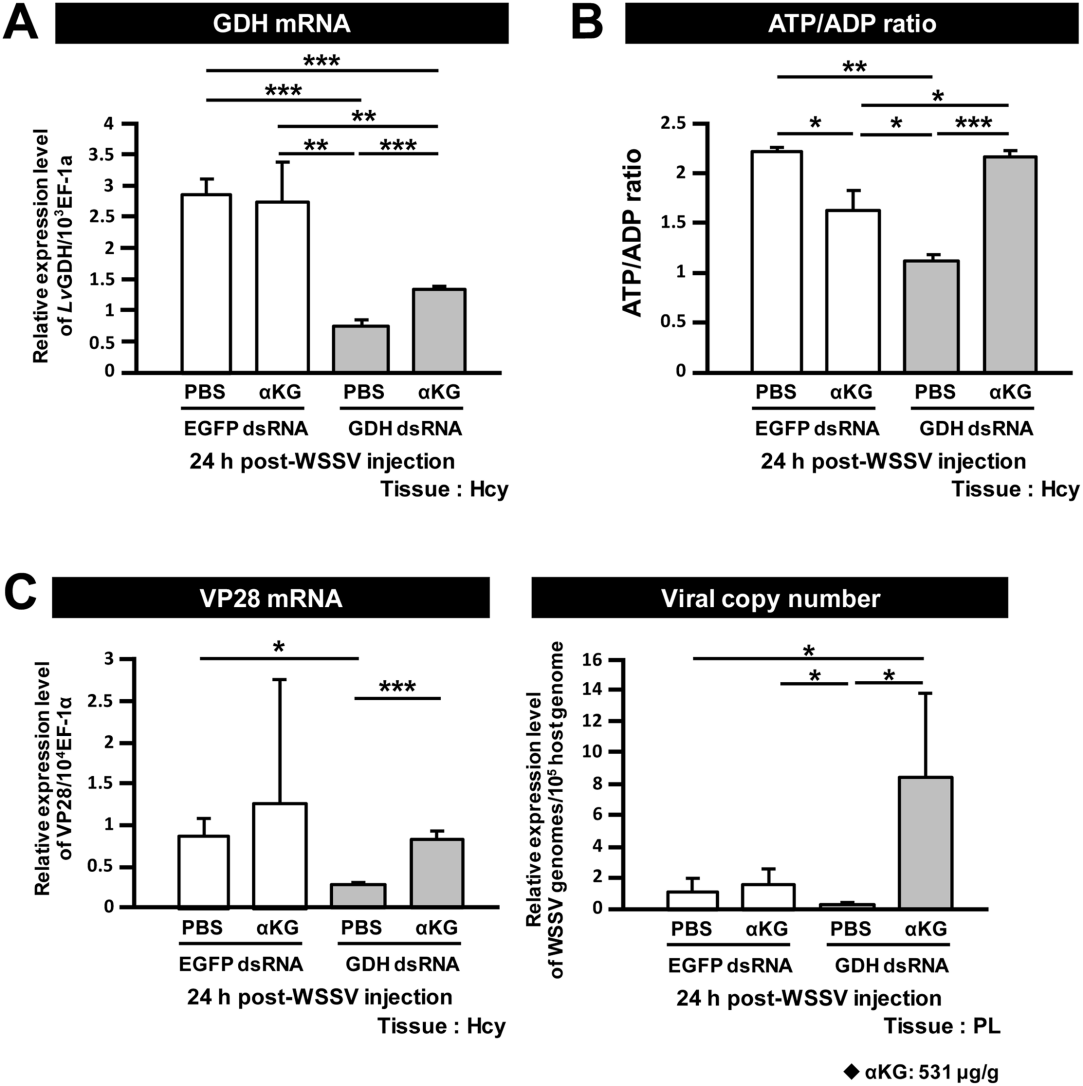
Glutamate-driven anaplerosis produces α-KG, which is an essential anaplerotic TCA cycle metabolite during WSSV infection. (A) GDH dsRNA treatment significantly decreased GDH expression. Three days after treatment with EGFP dsRNA and GDH dsRNA, shrimp were challenged with WSSV and then subjected to α-KG replenishment (531 μg/g shrimp) 2h post WSSV injection. At 24 h post WSSV injection, 4 hemocyte and pleopod samples (3 shrimp in each sample) were collected from each group. The hemocyte samples were used to measure mRNA expression and ATP/ADP ratio, while the pleopod samples were used to measure the viral copy number. Asterisks indicate significant differences between each group (Student’s t test, * *p*<0.05, ** *p*<0.01, *** *p*<0.001). (B) α-KG replenishment rescued the ATP/ADP ratio in the GDH dsRNA treated groups. Asterisks indicate significant differences between groups (Student’s t test, * *p*<0.05, ** *p*<0.01, *** *p*<0.001). (C) α-KG replenishment rescued both WSSV mRNA expression and WSSV genome copy number in the GDH dsRNA treated groups. Asterisks indicate statistically significant differences between each group (Student’s t test, * *p*<0.05, ** *p*<0.01, *** *p*<0.001).

Fig 5A shows that GDH mRNA was significantly reduced in both of the GDH dsRNA treated groups compared to the EGFP dsRNA groups. We note too that in the GDH silenced groups, injection of α-KG led to a small but significant increase in GDH mRNA levels (Fig 5A). The reason for this is not clear. In Fig 5B, if glutamate is converted into α-KG and enters the TCA cycle, then we could expect GDH silencing to result in a loss of ATP production. As expected, at 24 h post WSSV injection, there was a lower ATP/ADP ratio in the hemocytes collected from the GDH dsRNA group. Further, as predicted, the addition of α-KG indeed rescued the ATP/ADP ratio in the GDH-silenced shrimp after WSSV infection (Fig 5B). Moreover, WSSV mRNA expression and viral genome copy number in the GDH- silenced shrimp were both significantly increased after α-KG supplementation (Fig 5C). The ability of α-KG to rescue the effects produced by GDH silencing suggests that, after WSSV infection, glutamate is indeed converted to α-KG via GDH, and that the α-KG is used to replenish the TCA cycle to benefit WSSV replication.

## Discussion

In the present study, we have found that at the genome replication stage, in addition to the previously reported metabolic changes in glycolysis and lipolysis, WSSV-infected cells also induce a high uptake of glutamate, but not glutamine, from the hemolymph (Fig 1B and 1C). Our results further suggest that this leads to glutamate-driven anaplerosis (Fig 2C and 2D). Our experiments on dsRNA silencing of GDH and ASAT and α-KG replenishment (Figs 4 and 5), further showed that this WSSV-induced glutamate-driven anaplerosis is essential for WSSV replication. WSSV-induced anaplerosis would explain a previous observation that the metabolites which follow α-KG in the TCA cycle were significantly increased at 12 hpi, even while their respective enzymes, as well as the upstream metabolites acetyl-CoA, citrate and isocitrate, were either unchanged or down-regulated [5].

WSSV-induced glutamate-driven anaplerosis is unusual because unlike most cancer cells and virus-infected vertebrate cells, which boost glutaminolysis by increasing their uptake of glutamine [10, 13, 16, 27, 28], our data suggests that WSSV prefers to uptake glutamate as the carbon source (Fig 1B and 1C). At present, the lack of a shrimp cell line means that it is not possible to confirm this by amino acid limitation experiments (ie. by using medium that is free of either glutamine or glutamate), but we are currently developing a stable isotope labeling platform which will allow us to perform *in vivo* metabolic flux analysis in shrimp during WSSV infection. Meanwhile, we note that glutamate uptake commonly occurs in astrocytes to trigger glutamate signaling in neurometabolic coupling [29], and interestingly, as a consequence of this uptake, glutamate-stimulated aerobic glycolysis is subsequently triggered [30].

Glutamate is normally metabolized into α-KG via one of two pathways: (1) Direct reaction, in which glutamate is converted directly into α-KG in a reaction catalyzed by GDH; (2) Indirect reaction, in which the α amino group of glutamate is transferred to oxaloacetate to generate aspartate and α-KG in a reaction catalyzed by ASAT [16]. At the WSSV genome replication stage, our data shows an increase in the mRNA level of GDH (Fig 2B), an increase in the enzyme activities of both GDH and ASAT (Fig 2C), and also that GDH was up-regulated by the mTORC2 signaling pathway (Fig 3C and 3D). In addition, silencing of these two enzymes also led to significant decreases in the WSSV viral copy number (Fig 4). Because the conversion of glutamate by GDH and ASAT respectively produces different downstream metabolites, activation of one or both of these two enzymes will usually depend on the biological requirements of the cell [31, 32]. In the case of WSSV-triggered glutamate-driven anaplerosis, although only GDH mRNA was significantly upregulated at 12 hpi (Fig 2B), the importance and relative contribution of these two pathways still needs to be determined.

In cancer cells and virus-infected vertebrate cells, when anaplerosis of the TCA cycle is inhibited, cell growth and replication of the virus, respectively, are suppressed [15, 16, 27, 28, 33, 34]. Viral replication was also suppressed when the WSSV-induced anaplerosis was blocked by dsRNA silencing of GDH and ASAT: silencing led to significant decreases in WSSV mRNA expression and the number of WSSV genome copies (Fig 4), suggesting that the anaplerosis is crucial for WSSV replication. Anaplerosis allows the TCA cycle to continue to provide not only energy but also the intermediates for biosynthetic precursors, such as nucleic acids, amino acids and lipids [13, 18, 35]. It is known for instance that glutamine-derived α-KG is mainly used for lipogenesis during cell proliferation [18, 19]. However, although we have shown here that the ATP/ADP ratio was rescued in GDH-silenced shrimp by direct injection of the TCA cycle intermediate α-KG (Fig 5B), we have not yet been able to confirm that this anaplerosis is also important for the biosynthetic pathways that are necessary for WSSV to complete its replication cycle. Even so, we speculate that WSSV-induced glutamate-driven anaplerosis at the WSSV genome replication stage may generate the materials that are subsequently needed for lipogenesis at the late stage of WSSV replication (24 hpi; [25]). In particular, it will be interesting to investigate the role that WSSV-triggered glutamate-driven anaplerosis plays in the synthesis of the lipids that are used for the envelope fraction of the WSSV virion particles.

The PI3K-Akt-mTORC1 pathway is well-known as a key mechanism that drives the increase in glucose uptake and triggers the Warburg effect in the vast majority of cancer cells as well as in vertebrate cells infected by viruses such as HCMV and HPV [21, 36, 37, 38]. Although WSSV also relies on the activation of this pathway to trigger the Warburg effect, when we used LY294002 and Rapamycin to suppress PI3K and mTORC1, there was no reduction in GDH or ASAT expression at 12 hpi–and in fact mTORC1 suppression led to an increase in the mRNA levels of both enzymes (Fig 3B and 3C). This suggests that neither the PI3K- mTORC1 pathway nor mTORC1 alone are involved in triggering WSSV-induced glutamate-driven anaplerosis. This conclusion is consistent with other studies that have shown that, in some cases, glutaminolysis can be triggered independently of the PI3K-Akt-mTORC1 pathway [12, 39].

As noted above, when mTORC1 is suppressed, this can lead to the activation of mTORC2. mTORC2 can either compensate directly for the suppression of mTORC1 [26], or it can trigger other downstream responses independently of the PI3K-Akt-mTORC1 pathway [40], and these independent responses are now increasingly being recognized as important in cancer metabolic reprogramming [41]. Bearing these possibilities in mind, based on the Rapamycin and Torin 1 results for GDH (Fig 3C and 3D), while the exact role of mTORC1 is unclear–it may inhibit GDH expression, or it may not be involved at all–we infer that mTORC2 is in fact acting independently and that it somehow activates glutamate-driven anaplerosis at least at the transcriptional level. Thus in addition to our previous findings that mTORC2 was involved in the WSSV-induced Warburg effect and essential for successful WSSV genome replication [5], our present results now suggest that mTORC2 signaling is essential for WSSV-induced glutamate-driven anaplerosis. We further propose that mTORC2’s importance in WSSV replication is probably mediated by its regulation of this part of the glutaminolysis pathway. (Unfortunately, our attempts to confirm the respective roles that mTORC1 and mTORC2 play in WSSV replication *in vivo* have so far been unsuccessful because treatment with the appropriate dsRNAs have failed to silence the expression of raptor and rictor, which are key subunits of mTORC1 and mTORC2, respectively.)

**Fig 6:**
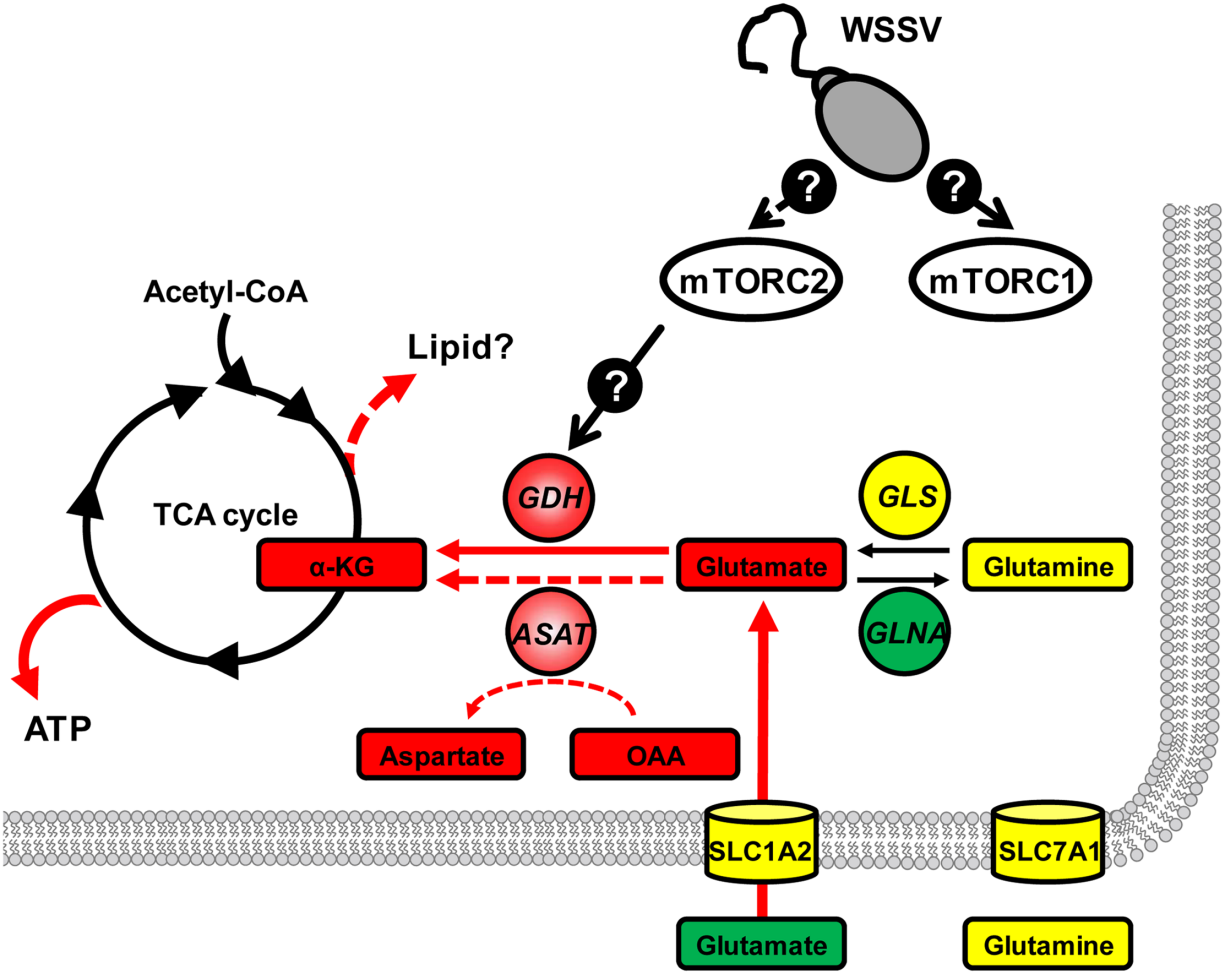
Proposed model of how WSSV-induced glutamate-driven anaplerosis is regulated by the mTORC2 at 12 hpi. Gene expression and/or protein levels of enzymes (circle) and the mRNA levels of transporters (cylinders) were either increased (red), decreased (green) or remained unchanged (yellow). Expression and protein data are compiled from the present study and from Su *et al*. [5]. The same color code is used for metabolites, which are shown in boxes. Except for α-KG, all metabolite data is from Su *et al* [5]. Details of the mTORC2 regulatory pathways are currently unknown. In addition to increasing energy levels (ATP), we speculate that replenishment of α-KG may also drive the production of lipid precursors (dashed red arrow).

Our proposed model of how WSSV triggers glutamate-driven anaplerosis at 12 hpi is shown in Fig 6. In this model, mTORC2 is activated after WSSV infection, and at the WSSV genome replication stage, mTORC2 signaling acts via unknown regulators to trigger the expression of GDH. At the same time, the excess of glutamate “pulled” from the hemolymph also serves to drive the conversion of glutamate to α-KG. This in turn provides anaplerotic replenishment of the TCA cycle, which ultimately supports virus replication.

## Supporting Information

S1 Fig GDH dsRNA has a specific, statistically significant silencing effect on GDH protein levels in WSSV-infected shrimp. (A)Western blotting results at 24 hpi for 3–4 samples of gill tissue taken from WSSV-infected shrimp that were treated with the indicated dsRNA (or PBS vehicle only control) 3 days prior to challenge. A commercial GDH antibody (Proteintech) was used as the probe. Signal strength was quantified using ImageJ software, normalized relative to β-actin, and then expressed relative to the positive control, which was set to 1. (B) Aggregated data and statistical analysis of the results shown in (A).
(DOCX)
