# Supplementary material for: Replication of the Shrimp Virus WSSV Depends on Glutamate-Driven Anaplerosis
Source: PLoS One. 2016 Jan 11;11(1):e0146902. doi: 10.1371/journal.pone.0146902 (PMC4709008; doi:10.1371/journal.pone.0146902)
Supplement: S1 Fig — (A)Western blotting results at 24 hpi for 3–4 samples of gill tissue taken from WSSV-infected shrimp that were treated with the indicated dsRNA (or PBS vehicle only control) 3 days prior to challenge. A commercial GDH antibody (Proteintech) was used as the probe. Signal strength was quantified using ImageJ software, normalized relative to β-actin, and then expressed relative to the positive control, which was set to 1. (B) Aggregated data and statistical analysis of the results shown in (A). (DOCX) [file pone.0146902.s001.docx]

**S1 Fig. GDH dsRNA has a specific, statistically significant silencing effect on GDH protein levels in WSSV-infected shrimp.**

(A)Western blotting results at 24 hpi for 3-4 samples of gill tissue taken from WSSV-infected shrimp that were treated with the indicated dsRNA (or PBS vehicle only control) 3 days prior to challenge. A commercial GDH antibody (Proteintech) was used as the probe. Signal strength was quantified using ImageJ software, normalized relative to β-actin, and then expressed relative to the positive control, which was set to 1. (B) Aggregated data and statistical analysis of the results shown in (A).

**S1 Figure**
